# Development of a thermostable nanoemulsion adjuvanted vaccine against tuberculosis using a design-of-experiments approach

**DOI:** 10.2147/IJN.S159839

**Published:** 2018-06-26

**Authors:** Ryan M Kramer, Michelle C Archer, Mark T Orr, Natasha Dubois Cauwelaert, Elyse A Beebe, Po-wei D Huang, Quinton M Dowling, Alicia M Schwartz, Dawn M Fedor, Thomas S Vedvick, Christopher B Fox

**Affiliations:** Infectious Disease Research Institute, Seattle, WA, USA, ryan.kramer@idri.org

**Keywords:** adjuvant, lyophilization, tuberculosis, formulation development, design of experiments, controlled temperature chain, GRAS

## Abstract

**Background:**

Adjuvants have the potential to increase the efficacy of protein-based vaccines but need to be maintained within specific temperature and storage conditions. Lyophilization can be used to increase the thermostability of protein pharmaceuticals; however, no marketed vaccine that contains an adjuvant is currently lyophilized, and lyophilization of oil-in-water nanoemulsion adjuvants presents a specific challenge. We have previously demonstrated the feasibility of lyophilizing a candidate adjuvanted protein vaccine against *Mycobacterium tuberculosis* (*Mtb*), ID93 + GLA-SE, and the subsequent improvement of thermostability; however, further development is required to prevent physicochemical changes and degradation of the TLR4 agonist glucopyranosyl lipid adjuvant formulated in an oil-in-water nanoemulsion (SE).

**Materials and methods:**

In this study, we took a systematic approach to the development of a thermostable product by first identifying compatible solution conditions and stabilizing excipients for both antigen and adjuvant. Next, we applied a design-of-experiments approach to identify stable lyophilized drug product formulations.

**Results:**

We identified specific formulations that contain disaccharide or a combination of disaccharide and mannitol that can achieve substantially improved thermostability and maintain immunogenicity in a mouse model when tested in accelerated and real-time stability studies.

**Conclusion:**

These efforts will aid in the development of a platform formulation for use with other similar vaccines.

## Introduction

Lyophilization has been routinely used to increase the shelf-life and thermostability of inactivated, live attenuated, bacterial, or subunit vaccines.^1^,^2^ Maintenance of the cold chain is problematic for distribution of vaccines in the developing world, and high vaccine wastage due to improper storage is a substantial cost.^3^,^4^ The World Health Organization (WHO) has established a controlled temperature chain (CTC) program to allow for storage of thermostable vaccines outside of 2°C–8°C, with the goal of decreasing vaccine wastage, increasing the ease of vaccine transportation, and decreasing the costs associated with vaccination in the developing world.^3^–^6^ Additionally, the need for vaccine stockpiling to combat natural disasters would benefit from the development of formulations that do not require a cold storage.^7^,^8^ The meningitis A vaccine MenAfriVac™ has been successfully administered under CTC during mass immunization in West Africa,^9^ and other vaccines are undergoing evaluation for CTC.^5^,^10^ Several marketed vaccines are lyophilized to improve thermostability; however, to date, no licensed vaccines that contain adjuvant are lyophilized.^1^ Next-generation recombinant subunit vaccines often require adjuvants such as aluminum salts or oil-in-water emulsions for efficacy, and lyophilization of adjuvant formulations, including aluminum, and oil-in-water emulsion adjuvants presents additional challenges.^11^–^16^

There were ~10.4 million incident cases and an estimated 1.3 million deaths due to tuberculosis (TB) in 2015.^17^ BCG is the only approved vaccine against *Mycobacterium tuberculosis* (*Mtb*) and successfully prevents disseminated TB in children; however, BCG fails to adequately protect against *Mtb* infection and against development of TB in adults or adolescents, with estimates of efficacy ranging from 0 to 80%.^18^,^19^ A new vaccine against *Mtb* that successfully protects adults is needed to boost or replace BCG. Mathematical modeling predicts that implementation of a new vaccine with 60% efficacy would result in an 80% decrease in new TB cases by the year 2050.^20^ We have developed ID93 + GLA-SE as a vaccine against *Mtb* that is composed of a recombinant fusion of four *Mtb* protein antigens (ID93) and the TLR4 agonist glucopyranosyl lipid adjuvant (GLA) formulated in an oil-in-water emulsion adjuvant (GLA-SE).^21^–^23^ The vaccine elicits a TH1 response and confers protective efficacy against *Mtb* in experimental models. This vaccine has successfully completed Phase I safety studies in the US and South Africa and is currently undergoing a Phase II study to assess prevention of TB recurrence. These clinical studies utilize a two-vial presentation with the rehydrated lyophilized antigen and liquid adjuvant mixed in the clinic prior to administration. A single-vial thermostable formulation would greatly aid in distribution and reduce shipping costs associated with implementing a new *Mtb* vaccine.

We have previously shown that ID93 + GLA-SE can be successfully lyophilized and both the antigen and adjuvant preserved their physicochemical properties after reconstitution.^24^ Additionally, after storage at 50°C for 30 days, the vaccine remained immunogenic and retained protective efficacy in a mouse model of pulmonary *Mtb* infection; however, partial GLA degradation and moderate particle size increases were seen due to thermal sensitivity of GLA-SE when lyophilized in the same vial with ID93. This paper represented the first published lyophilization and biophysical characterization of a nanoemulsion adjuvanted vaccine. However, further development is desired to achieve a more thermostable product. Herein, we describe formulation development efforts to advance a single-vial thermostable presentation intended for future clinical testing. Our preformulation approach included separate antigen and adjuvant physicochemical characterization and excipient screening studies in the liquid state to identify stabilizing or compatible excipients, pH, and buffers for the antigen and adjuvant. We subsequently selected six excipients based on their compatibility with ID93 and GLA-SE in liquid solution and/or for their anticipated utility in lyophilization. We then utilized a design-of-experiments (DoE) approach to identify optimized excipient compositions for the lyophilized ID93 + GLA-SE vaccine based on their ability to maintain antigen and adjuvant stability following thermal stress at 50°C for 30 days. From the lyophilized formulation development studies, six lead compositions were selected and subsequently tested in longer-term storage stability and immunogenicity studies.

## Materials and methods

### Sample preparation for physicochemical characterization

ID93 in 20 mM Tris pH 8 buffer was produced and purified at IDRI and stored at −80°C prior to use. For the initial solubility screen, protein was diluted with 20 mM citrate-phosphate buffer (pH 3–8), with or without the addition of NaCl (*I* = 0.15) as indicated in the text, to a final ID93 concentration of 0.2 mg/mL. Samples were held at room temperature for 2 hours to allow for instability-indicating precipitation to occur. All samples were then centrifuged for 10 minutes at 17,000 × g, and the supernatant was collected for UV analysis to determine protein concentration. Subsequent studies were performed using bulk ID93 dialyzed into 1 L of 20 mM citrate-phosphate buffer (pH 6.5–8.5, no NaCl) using 10K molecular weight cut-off (MWCO) Slide-A-Lyzer dialysis cassettes (Thermo Fisher Scientific, Waltham, MA, USA). Samples were diluted to 0.2 mg/mL using 0.22 µm sterile-filtered dialysate prior to analysis.

### Far-UV circular dichroism

Circular dichroism (CD) measurements were performed using a Jasco J-815 Spectropolarimeter (Jasco, Oklahoma, OK, USA) equipped with a Peltier temperature controller. A 2 mm path-length quartz cuvette (Starna Cells Inc., Atascadero, CA, USA) was loaded with 0.4 mL of 0.2 mg/mL sample or buffer and sealed with a Teflon stopper. Complete spectra from 250 to 190 nm were collected at 10°C and 90°C, and discrete wavelengths (208, 218, and 222 nm, using a 1 nm bandwidth) were monitored every 2°C, ramping from 10°C to 90°C with a temperature ramp rate of 0.7°C/min and a 4-second data integration time. Samples were measured in duplicate, and data were analyzed using OriginPro (V 9.0) software after blank subtraction with the buffer.

### Intrinsic fluorescence and right-angle static light scattering

A Cary Eclipse fluorescence spectrometer (Agilent Technologies, Santa Clara, CA, USA) was used to collect emission spectra (305–405 nm) and right-angle static light scattering (295 nm) of samples excited with 295 nm light (5 nm slit width). One-centimeter square quartz cuvettes (Starna Cells Inc.) with four polished sides and interior dimensions of 2 × 10 mm were loaded with 0.5 mL of 0.2 mg/mL sample or buffer and sealed with a Teflon stopper. Samples were heated from 10°C to 90°C with measurements at 2°C increments. A scan rate of 60 nm/min, an averaging time of 1.0 second, a data interval of 1 nm, and a photomultiplier tube detector voltage of 550 V were used. Samples were measured in duplicate, and data were analyzed using OriginPro (V 9.0) software after blank subtraction with the buffer.

### Turbidity measurements

A Cary 100-UV-Visible spectrophotometer (Agilent Technologies), equipped with a 12-position Peltier temperature controller, was used to monitor increases in sample turbidity by following the optical density at 350 nm (bandwidth of 1 nm). One-centimeter quartz cuvettes (Starna Cells Inc.) were loaded with 0.2 mL of sample or buffer. Data were collected every 1°C for 3 seconds from 10°C to 90°C with a temperature ramp rate of 0.2°C. For excipient screening, a high-throughput turbidity-based kinetic assay was performed. Samples or buffer were held at 53°C for 120 minutes, and data were collected for 0.1 seconds every 30 seconds. Samples were measured in duplicate, and data were analyzed using OriginPro (V 9.0 and V 9.1) software after blank subtraction with the buffer.

### Dynamic light scattering measurements

Particle mean hydrodynamic diameter (Z-average diameter) and polydispersity index were measured by dynamic light scattering (DLS) at a 90° angle using a Malvern Zetasizer APS (Malvern, UK) as has been previously described.^25^ Samples were diluted 1:10 in Milli-Q water in a 96-well plate and mixed gently with a pipette. Three measurements were made for each sample. For GLA-SE excipient compatibility studies, diluted excipient without GLA-SE was also measured to confirm that changes in the particle size can be attributed to interactions between the emulsion and the excipient rather than particles in the excipient.

### GLA quantitation by reversed-phase HPLC

Reversed-phase HPLC analysis of GLA was performed on an Agilent 1200 series HPLC (Agilent Technologies). Separation was performed on a C18 Atlantis T3 reversed-phase column (Waters, Milford, MA, USA) with a gradient over 30 minutes. Mobile phase A contained 75:15:10 (v/v/v) methanol:chloroform:water, 1% (v/v) acetic acid, and 20 mM ammonium acetate. Mobile phase B contained 50:50 (v/v) methanol:chloroform, 1% (v/v) acetic acid, and 20 mM ammonium acetate. Column temperature was held constant at 30°C, and GLA was detected with a Corona Charged Aerosol Detector (CAD) (ESA Biosciences, Chelmsford, MA, USA). Triplicate samples were diluted 1:50 in mobile phase B and analyzed using a nine-point GLA standard curve prepared in mobile phase B. The peak areas from the standards were determined by integration, and the peak areas were fit with a second-order polynomial. GLA content of samples was calculated by interpolation.

### ID93 quantitation by reversed-phase HPLC with fluorescence detector

Reversed-phase HPLC analysis of ID93 was performed on an Agilent 1100 series HPLC and a fluorescence detector (Agilent Technologies). Separation was performed using a 4.6 × 150 mm, 3.5 µm XBridge protein BEH C4, 300 Å analytical column (Waters). Mobile phase A contained 0.1% TEA in water, and mobile phase B contained 0.1% TEA in acetonitrile. A linear gradient was generated at 1.2 mL/min: 0–30 minutes, 2%B–80%B; 30–31 minutes, 80%B–2%B; and 31–35 minutes, 2%B. The column temperature was held at 60°C. A four-point standard curve containing ID93 and GLA-SE (1% oil, 25 µg/mL) was used. SDS and BME were added to the sample for final concentrations of 5% and 0.6% (w/v), respectively. All standards and samples were run in duplicate. Fluorescence emission intensity was collected at 350 nm with excitation at 280 nm.

### Empirical phase diagram

An empirical phase diagram (EPD) was generated for ID93 using CD molar ellipticity at 208 nm, right-angle static light scattering intensity, and fluorescence emission spectra mean spectral mass wavelength as a function of temperature (10°C–90°C) and pH (6.5–8.5). The EPD was generated using MATLAB software (The MathWorks, Inc., Natick, MA, USA) as has been described previously.^26^ In short, the variables from each technique are arranged into parameter sets (pH, temperature), and the sets of variables are in an n-dimensional vector space, where n is the number of variables in each set. Unit vectors are used to obtain a density matrix. The three largest components of the eigenvalues and eigenvectors of the density matrix are represented as color components in an RGB-based scheme. The three color components are summed to obtain a final color that represents that physical state of the protein at a particular pH and temperature. Different colors correspond to different states of the protein.

### Sample preparation for excipient screening

Excipients were prepared at a 2X concentration containing 20 mM Tris. Solutions were pH adjusted with sodium hydroxide or hydrochloric acid to pH 7.0 ± 0.05. Excipient solutions were then passed through a 0.22 µm PES membrane sterile filter. ID93 was dialyzed using 10K MWCO Slide-A-Lyzer dialysis cassettes into 1 L of 20 mM Tris buffer pH 7.0 ± 0.05 at 4°C overnight. Samples were diluted to 0.2 mg/mL using 0.22 µm sterile-filtered dialysate prior to analysis.

### GLA-SE excipient compatibility study

GLA-SE excipient compatibility was screened in a 96-well plate format. First, GLA-SE was diluted from stock concentration (10% [v/v] oil, 0.25 mg/mL GLA) to a 2X working concentration (4% oil, 0.1 mg/mL GLA) in 25 mM ammonium phosphate buffer pH 5.5. Then, a 96-well plate was loaded with 100 µL of working concentration GLA-SE, and each well was mixed 1:1 with 2X excipient. Wells were mixed by pipetting up and down repeatedly. The plate was incubated for 1 hour at 25°C before samples were collected for analysis by DLS and HPLC. The plate was returned to the incubator. After 24 hours, a second set of samples was collected for analysis. Visual observations were also recorded. At each time point, a clean pipette tip was drawn through each well to visually check for creaming or settling.

### Sample preparation for DoE and stability studies

Samples were prepared with 0.1 mg/mL or 5 µg/mL ID93, 2% oil, 0.05 mg/mL GLA, and 1X excipient in 20 mM Tris pH 8.0, Tris pH 7.5, sodium phosphate pH 8.0, or sodium phosphate pH 7.5 ± 0.05. For each formulation, six 3 mL glass vials were filled with 1 mL of sample. Of these six vials, one was set aside for liquid pre-lyophilization characterization, while the remaining five sample vials were lyophilized.

### Sample lyophilization and reconstitution

Lyophilization was performed in a Virtis AdVantage 2.0 EL-85 bench-top freeze dryer (SP Scientific, Warminster, PA, USA) as described elsewhere.^27^ Briefly, the freeze–dry cycle included a 2-hour freeze at −50°C followed by a 36-hour primary drying step at −30°C and a 10-hour secondary drying step at 25°C with vacuum applied at 50 mTorr. At the completion of the cycle, samples were brought back to atmospheric pressure, blanketed with high-purity nitrogen, and stoppered prior to being removed from the freeze-dryer chamber. Five vials of each formulation were lyophilized. Of the five, one vial was used immediately for post-lyophilization characterization, and the remaining vials were crimped with aluminum seals. One crimped vial was placed in a 50°C incubator to simulate an accelerated stability or “stressed” condition, and the remaining three crimped vials were stored at 4°C. Reconstitution of samples was performed by removing the stoppers from the vials and adding 1 mL of Milli-Q water using a pipette. The stopper was replaced, and the sample was swirled until fully reconstituted.

### DoE and analysis of results using JMP software

JMP Version 11.2.0 was used for all DoE and analysis. The custom design option was used to create I-optimal designs for each DoE.^28^ Three designs were generated using factors as described in the “Optimization of formulations using DoE” section. Designs were selected with the goal of optimizing excipient identity and concentrations, and modeling excipient effects and interactions. Specifically, models included terms to estimate main effects, and second-order, third-order, and second-power interactions. Optimization and modeling was performed using a least squares fit on particle size, GLA concentration, pH, and cake quality. Cake quality was encoded numerically where 1 is an acceptable or excellent cake with no change in appearance when stressed and 2 is a poor-quality cake after lyophilization, or a cake that changed when heat stressed. JMP creates a prediction profiler for each response from the least squares fit. Desirability functions were assigned based on our target product profile for each response. The functions were, broadly, as follows: particle size was minimized, GLA concentration was maximized, change in pH was minimized, and poor cake quality was minimized. Specific desirability settings are shown in Table S1. Responses were given equal importance in the desirability function. The optimum formulations were then selected by maximizing overall desirability.

### Visual assessment of lyophilized cake quality

Visual observations were performed on cakes post-lyophilization and post-stress at 50°C. Cakes were assessed by structure, shape, and uniformity within a sample set (consisting of five vials per formulation). Upon lyophilization, cakes were ranked as “excellent”, “acceptable”, and “poor” as defined by characteristics relative to other cakes in the entire study. “Excellent” cakes were dense and retained the same volume as the original fill volume. Small cracks, peaks, and nonuniform features may exist. “Poor” cakes were severely shrunken and/or had nonuniformity within its sample set. “Acceptable” cakes were all cakes in between “excellent” and poor”. Characteristics of these cakes included uniform moderate shrinkage, varying shrinkage from the top to the bottom of a cake, and the presence of superficial or deep cracks. After stress, cakes were then classified as exhibiting “no change” or “change”. Cakes with “no change” were identical in appearance to post-lyophilization without heat stress. Cakes with “change” showed minor changes, such as uniform shrinkage no more thañ1 mm around the edge of the cake, to major changes, such as severe shrinkage or collapse.

### Mouse immunogenicity experiments

For immunogenicity experiments, 6- to 8-week-old female C57BL/6 mice were purchased from The Jackson Laboratory and maintained in specific pathogen-free conditions. All animal study protocols were approved by the IDRI Institutional Animal Care and Use Committee (IACUC) and were performed according to the IACUC regulations and guidelines. Mice were immunized two times 3 weeks apart by intramuscular injection of 100 µL of the indicated vaccine preparation. Mouse sera (N = 5/group) were prepared 28 days after the second immunization by collection of retro-orbital blood into microtainer serum collection tubes (VWR International, Radnor, PA, USA), followed by centrifugation at 1,062 × g for 5 minutes. Each serum sample was then analyzed by antibody capture ELISA. Briefly, ELISA plates (Nunc, Rochester, NY, USA) were coated with 2 µg/mL ID93 in 0.1 M bicarbonate buffer and blocked with 1% BSA-PBS. Then, in consecutive order and following washes in PBS/Tween 20, serially diluted serum samples, anti-mouse IgG, IgG1, or IgG2c-HRP (all from Southern Biotech, Birmingham, AL, USA), and ABTS-H_2_O_2_ (Kirkegaard and Perry Laboratories, Gaithersburg, MD, USA) were added to the plates. Plates were analyzed at 405 nm (EL_X_808; Bio-Tek Instruments Inc, Winooski, VT, USA). One month after the final immunization, splenocytes were isolated from five animals per group. Red blood cells were lysed using Red Blood Cell Lysis Buffer (eBioscience, San Diego, CA, USA) and resuspended in RPMI 1640 (Gibco; Thermo Fisher Scientific) and 10% heat-inactivated FBS (Sigma-Aldrich, St Louis, MO, USA). Cells were plated at 2 × 10^6^ cells/well in 96-well U-bottom plates (BD Biosciences, San Jose, CA, USA) and were stimulated for 1 hour with media or ID93 (10 µg/mL) at 37°C. Then, 1 µg/mL BrefeldinA (GolgiPlug; BD Biosciences) was added, and the cells were incubated for an additional 7 hours at 37°C. Cells were washed and surface stained with fluorochrome-labeled antibodies to CD4 (clone GK1.5), CD8 (clone 53-6.7), and CD44 (clone IM7) (BioLegend, San Diego, CA, USA) in the presence of anti-CD16/32 (clone 2.4G2) for 20 minutes at room temperature. Cells were washed and permeabilized with Cytofix/Cytoperm (BD Biosciences) for 20 minutes at room temperature. Cells were washed twice with Perm/Wash (BD Biosciences) and stained intracellularly with fluorochrome-labeled antibodies to IFN-γ (clone XMG-1.2), IL-2 (JES6-5H4), TNF (MP6-XT22), CD154 (clone MR1), IL-5 (clone TRFK5), and IL-17A (clone TC11-18H10.1) (BioLegend) for 20 minutes at room temperature. Cells were washed and resuspended in PBS + 1% BSA. Up to 10^6^ total events were collected on a four-laser LSRFortessa flow cytometer (BD Biosciences). Data were analyzed with FlowJo. Cells were gated as singlets > lymphocytes > CD4+CD8− > CD44+ > cytokine positive. ID93-specific response frequencies were determined by subtracting the frequency of response positives of unstimulated cells from ID93-stimulated cells in matched samples from the total CD4+CD8− population.

## Results and discussion

### Physicochemical characterization

**Figure 1:**
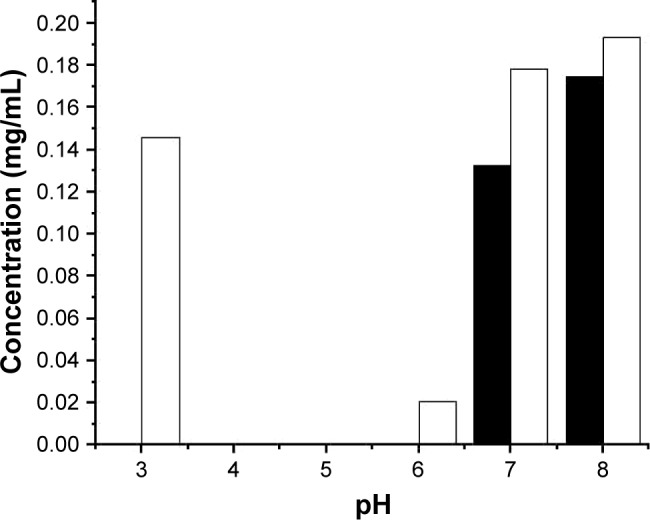
ID93 remaining in solution after centrifugation subsequent to mixing with the 20 mM citrate-phosphate buffer at the indicated pH with (filled bars) or without (open bars) the addition of NaCl to increase the ionic strength to *I* = 0.15. Results shown are from a single experiment.

ID93 was mixed with citrate-phosphate buffers from pH 3 to 8, with or without the addition of sodium chloride, to obtain 0.2 mg/mL samples. Sample concentration was determined subsequent to centrifugation (Figure 1). In the absence of sodium chloride, loss of protein was detected from pH 4 to 6. Loss of protein was exacerbated with the addition of 150 mM sodium chloride and was observed from pH 3 to 7, suggesting that NaCl lowers the solubility of ID93 and limits the pH range suitable for formulation. Based on these observations, citrate-phosphate buffer without sodium chloride (pH 6.5–8.5) was utilized for the physicochemical analysis of ID93. This buffer and pH range were used to study the secondary structure, tertiary structure, and aggregation of the protein to identify stable formulation conditions.

**Figure 2:**
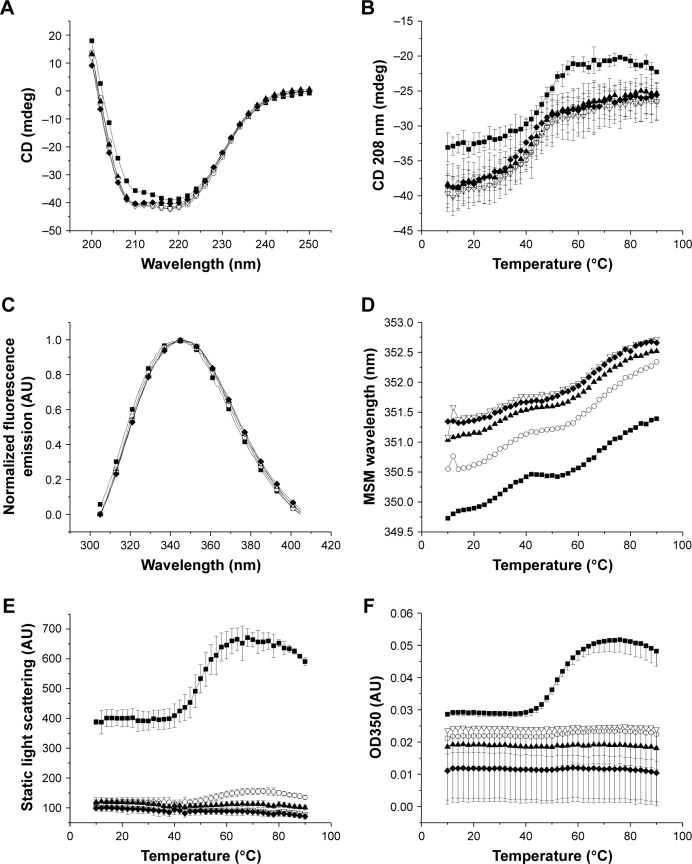
Physicochemical data collected for ID93 as a function of temperature (10°C–90°C) and pH (6.5–8.5). Secondary structure of ID93 was assessed by CD at 10°C from 200 to 250 nm (**A**) and monitored at 208 nm as a function of temperature (**B**). Tertiary structure of ID93 was assessed by intrinsic fluorescence, and the emission spectra were collected from 305 to 405 nm after excitation at 295 nm. Normalized fluorescence emission spectra are shown at 10°C (**C**), and the mean spectral mass was determined at each temperature (**D**). Aggregation of ID93 was monitored by static light scattering (**E**) and OD350 nm measurements (**F**). Data are shown for pH 6.5–8.5 (filled squares, open circles, filled triangles, open triangles, and filled diamonds 0.5 pH increments, respectively). Data were collected in duplicate, and error bars represent the standard deviation of those measurements. **Abbreviations:** CD, circular dichroism; MSM, mean spectral mass.

To monitor the secondary structure of ID93, far-UV CD was followed as a function of temperature and pH (10°C–90°C, pH 6.5–8.5). All CD spectra obtained at 10°C (Figure 2, Panel A) demonstrated broad minima and indicated mixed alpha–beta conformations. Slightly lower CD signal was observed for pH 6.5. Loss of secondary structure was followed at 208 nm (Figure 2, Panel B), and similar transition shapes and temperatures were observed for pH 7–8.5 with *T*_onset_ and *T*_m_ values of ~30°C and 45°C, respectively. For pH 6.5, higher *T*_onset_ and *T*_m_ values were observed.

The 13 tryptophan residues of ID93 were utilized as intrinsic probes of tertiary structure as a function of pH and temperature. Changes in the tryptophan microenvironments were monitored by selectively exciting tryptophan at 295 nm and following the emission mean spectral mass. Emission spectra maxima varied from 343 to 345 nm at 10°C, indicative of partially solvent-exposed tryptophan residues (Figure 2, Panel C). Emission maxima occurred at shorter wavelengths for pH 6.5 and 7, indicating less solvent exposure. Two distinct red-shift transitions were observed with increasing temperature (Figure 2, Panel D) signifying further solvent exposure of tryptophan and loss of tertiary structure if temperatures were increased.

Aggregation of ID93 was examined by right-angle light scattering (Figure 2, Panel E) and OD350 nm measurements (Figure 2, Panel F). Right-angle light scattering data were collected by monitoring the excitation wavelength during the fluorescence emission experiment. Light scattering intensity increased with particle size and concentration. Subvisible aggregates were evident at pH 6.5, even at low temperature, with a transition onset observed at 44°C–46°C by both right-angle light scattering and OD350 nm measurements. A lower-intensity transition was observed at approximately the same temperature for pH 7.0. No aggregation was observed for pH 7.5–8.5 from 10°C to 90°C.

**Figure 3:**
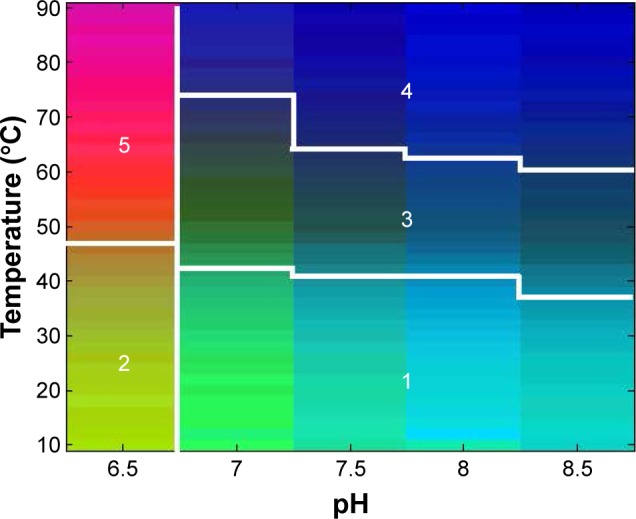
An EPD of ID93 using the physicochemical data from Figure 2. At least five distinct phases are evident. **Abbreviation:** EPD, empirical phase diagram.

The physicochemical data generated in the preceding section were used to generate an EPD (Figure 3).^26^ At least five distinct phases are evident in Figure 3. The properties of each phase can be identified by referring to the experimental data. Phase 1 is representative of the structural properties of ID93 at low temperatures from pH 7 to 8.5 and exhibits mixed alpha–beta structure and a greater degree of tertiary structure than at higher temperatures. Phase 2 is similar to phase 1 but is distinct in the level of aggregation observed by right-angle light scattering and turbidity measurements. Phase 3 represents an unfolding intermediate, and phases 4 and 5 exhibit greater levels of unfolding. Evident in the phase diagram, though not assigned a distinct phase, is the minor aggregation and fluorescence emission blue shift observed at pH 7. A phase boundary is observed between phases 1 and 3 at ~42°C–36°C, with the temperature of the phase boundary decreasing with increasing pH. This indicates that formulating at pH 7.5 or 8 may achieve a balance between minimizing aggregation and maximizing the temperature at which unfolding occurs.

### Excipient screening

**Figure 4:**
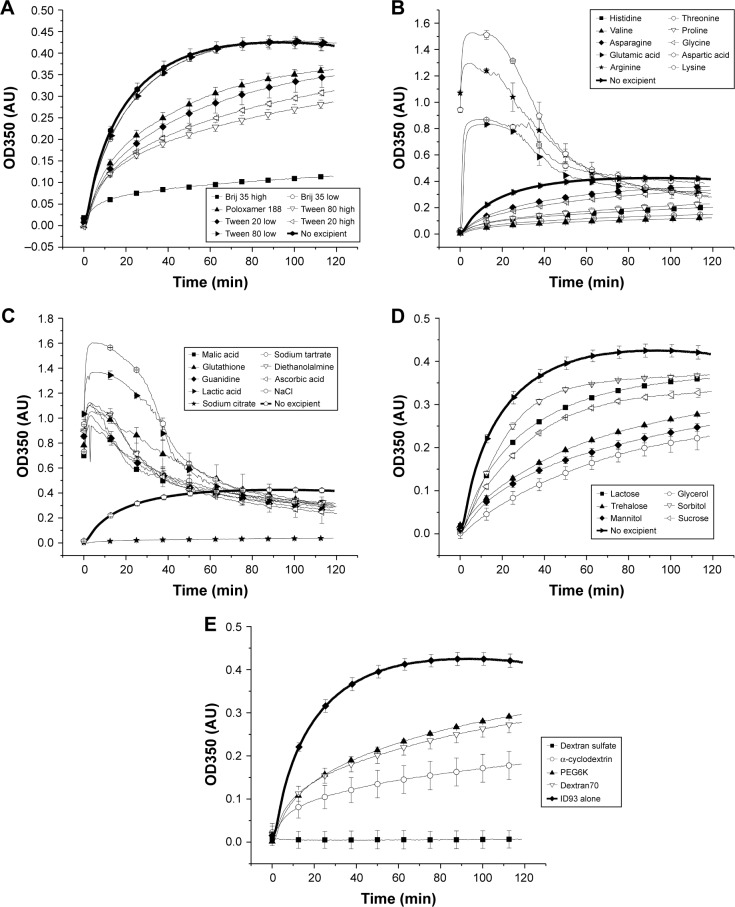
Aggregation of ID93 as a function of temperature. Excipients are grouped as detergents (**A**), amino acids (**B**), charged compounds (**C**), sugars and sugar alcohols (**D**), and other (**E**). ID93 is shown as a bold curve in each figure for comparison. Data were used to calculate the maximum OD350 values used in Figure 5 and Table 1.

**Figure 5:**
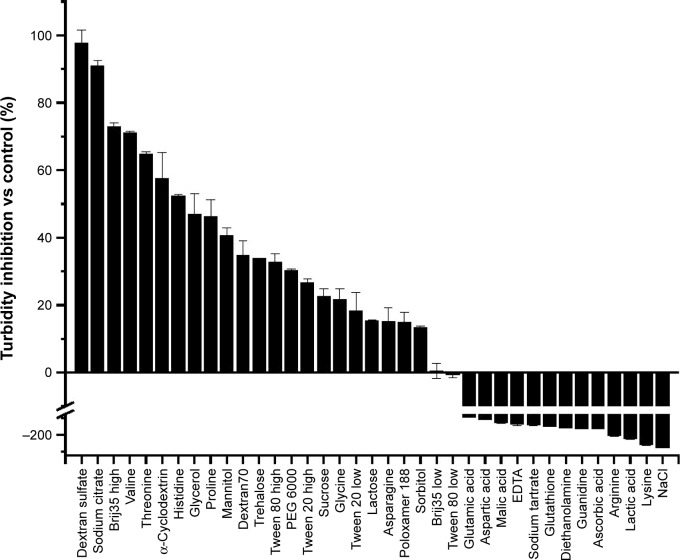
Percent turbidity inhibition of ID93 by the indicated excipient in 20 mM Tris pH 7.0 buffer. Table 1 provides the excipient concentrations. Percent turbidity inhibition is calculated from 1 minus the ratio of maximum OD350 of ID93 with excipient and the maximum OD350 of ID93 alone, and converting to percent. **Abbreviation:** PEG, polyethylene glycol.

A high-throughput, turbidity-based screening assay was developed to evaluate a library of excipients for potential stabilizers of ID93. During physicochemical characterization, increased aggregation of ID93 was observed at and below neutral pH, while the conformational stability decreased with increasing pH. Ionic strength was also determined to increase the level of aggregation observed, and the solubility of ID93 was decreased. Therefore, we sought to identify excipients which stabilized ID93. A kinetic assay was developed to screen excipients in the presence of 20 mM Tris buffer, pH 7.0, and aggregation was monitored by OD350 for 120 minutes at 53°C (Figure 4). The conditions of this assay were selected such that aggregation could be monitored; however, the physicochemical data suggested that formulation at pH 7.5 or 8 may be preferable to pH 7. This assay was used to screen a library of 37 excipients for stabilizers of ID93. Excipients were selected from the GRAS list, the FDA list of inactive ingredients in approved drug products, and additional research excipients were included. The excipients and concentrations studied as well as results from the screening assay are listed in Table 1. The maximum OD350 nm (Max OD_350_) values are listed and the data plotted as percent inhibition of aggregation in Figure 5. Seventeen excipients were found that inhibited the maximum aggregation by more than 20% and 10 that inhibited by more than 40%. Thirteen excipients that substantially increased the aggregation of ID93 were identified by this screen, and all of these substantially increased the solution ionic strength, further implicating high ionic strength as negatively impacting the stability of ID93 to aggregation. Plots of the results from the kinetic assay as optical density at 350 nm vs time are shown in Figure 4. Detergents (Figure 4A) tested below their critical micelle concentration (CMC) generally had minimal effect on the aggregation of ID93, whereas detergents tested above the CMC exhibited a greater inhibition of aggregation. Brij 35 and polysorbate 80 at 0.1% (w/v) demonstrated the largest inhibition. Amino acids (Figure 4B) with charged side chains (aspartic acid, glutamic acid, lysine, and arginine) increased the aggregation observed, while the amino acids with uncharged side chains (valine, proline, glycine, histidine, asparagine, and threonine) inhibited aggregation. Other charged or ionizable compounds similarly increased aggregation levels (Figure 4C). Sodium citrate, which was tested at 0.01 M due to the propensity to cause injection-site pain at higher concentrations, significantly inhibited the aggregation of ID93. All sugars and sugar alcohols (Figure 4D) tested were observed to inhibit aggregation. The largest effects were seen for glycerol, mannitol, trehalose, and sucrose. Other excipients screened that inhibited aggregation (Figure 4E) include dextran T70, dextran sulfate, polyethylene glycol 6000, and α-cyclodextrin.

The excipients were tested for compatibility with GLA-SE. Z-average particle size and GLA concentrations were determined after 1 and 24 hours of incubation at 25°C. Particle size changes of less than 5 nm from a starting size of 91.6 ± 0.9 nm were observed for all conditions with the exception of dextran sulfate and α-cyclodextrin (Table 1). The α-cyclodextrin-containing sample increased in size to over 200 nm, and sedimentation was visually observed. All other samples appeared visually similar to GLA-SE. An increase in particle size was also observed for the sample containing dextran sulfate; however, this was likely due to the large size of the excipient alone. This change in size was not evident in the number percentage distribution, indicating a minor population of large particles. GLA concentration was also measured 1 hour and 24 hours after mixing, and no change in GLA concentration was observed (data not shown). In summary, the particle size, visual observation, and GLA concentration data indicate broad compatibility of GLA-SE with the excipients tested in this study. Only α-cyclodextrin demonstrated incompatibility; however, formulations containing dextran sulfate may be more difficult to characterize.

### Optimization of formulations using DoE

Six excipients were selected for further study: trehalose, sucrose, mannitol, glycine, polysorbate 80, and sodium citrate. Excipients that displayed the greatest inhibition of aggregation within a given class of excipients or that are more commonly utilized in lyophilization were selected. α-Cyclodextrin was excluded from this portion of the study due to incompatibility with GLA-SE, dextran sulfate was excluded due to analytical challenges associated with measuring GLA-SE particle size, Brij 35 was excluded due to causing conformational disruptions to ID93 (data not shown), and glycine was selected over other amino acids due to utility in lyophilization. Additionally, Tris and sodium phosphate buffers at pH 7.5 and 8.0 were identified to provide conditions that resulted in both increased conformational stability and reduced propensity for aggregation. These conditions were utilized as factors for three separate DoE studies to optimize lead formulations of lyophilized ID93 + GLA-SE. Three DoE studies were constructed using the custom design option in JMP^®^. This design allows for the inclusion of continuous and categorical factors. The factors and ranges investigated in the three DoE studies are summarized in Table 2. The first DoE (DoE1) included the following factors: disaccharide type (trehalose or sucrose) and concentration (3.5%–10%, w/v), mannitol concentration (0–1%, w/v), buffer type (20 mM Tris or sodium phosphate), and pH (7.5 or 8.0). The primary objective of DoE1 was to optimize disaccharide–mannitol formulations (Table S2). The maximum concentration of mannitol was set to 1% (w/v) to encourage formation of an amorphous solid phase, since formation of a crystalline lyophilisate was observed to increase GLA-SE particle size after reconstitution (unpublished work). Additionally, a minimum concentration of 3.5% (w/v) of disaccharide and a constraint to restrict the minimum sample tonicity to 0.5X isotonic to human blood were used to minimize low-tonicity samples. The secondary objective of DoE1 was to investigate the role of buffer type (Tris or sodium phosphate) and pH (7.5 or 8.0). DoE2 factors included glycine and mannitol concentration (0–1%, w/v) in 20 mM Tris pH 8.0 (Table S3). The concentration range of each excipient was set to 0–1% (w/v) to promote the formation of an amorphous solid phase. Only Tris pH 8.0 buffer was used for this study to minimize sample number; however, alternative buffers could be selected for further study if glycine–mannitol formulations were identified as stabilizing or if another buffer was shown to be preferred in DoE1. DoE3 factors were disaccharide type (trehalose or sucrose) and concentration (2.5%–10%, w/v), and glycine concentration (0–1%, w/v), in a 20 mM Tris pH 8.0 buffer (Table S4). In addition to the three DoE studies, the effect of two additives, sodium citrate and Tween 80, were evaluated in select samples (Table S5). DoE1 contained 40 samples including five center points and five replicates, DoE2 contained 12 samples including one center point and three replicates, and DoE3 contained 15 samples including one center point and three replicates. The number of samples was such that the parameters for main effects, and second-order, third-order, and second-power terms could all be estimated.

**Figure 6:**
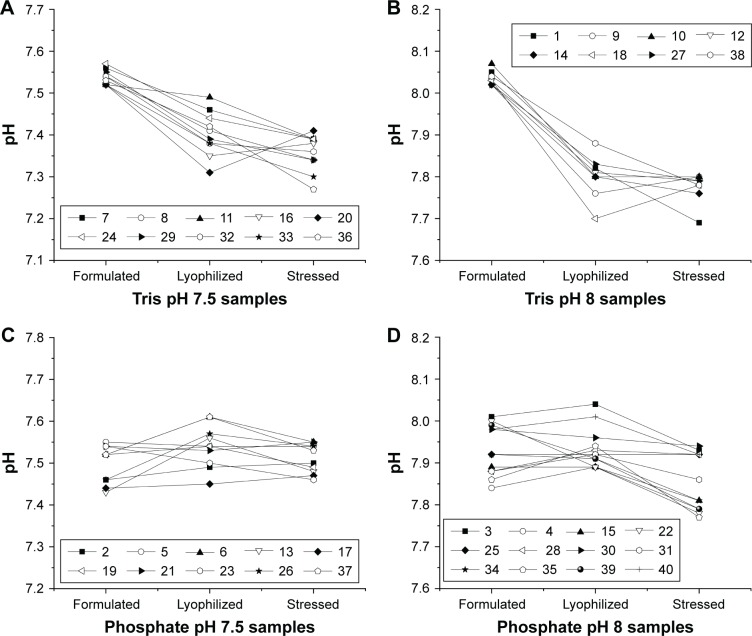
pH of samples from DoE1 measured after formulation, lyophilization and reconstitution, and after heat stress. Samples are arranged by buffer type and pH including Tris pH 7.5 (**A**), Tris pH 8.0 (**B**), phosphate pH 7.5 (**C**), and phosphate pH 8.0 (**D**). The largest changes in pH are seen for Tris buffer after lyophilization, whereas smaller changes are seen for phosphate buffers (see Table S2).

**Figure 7:**
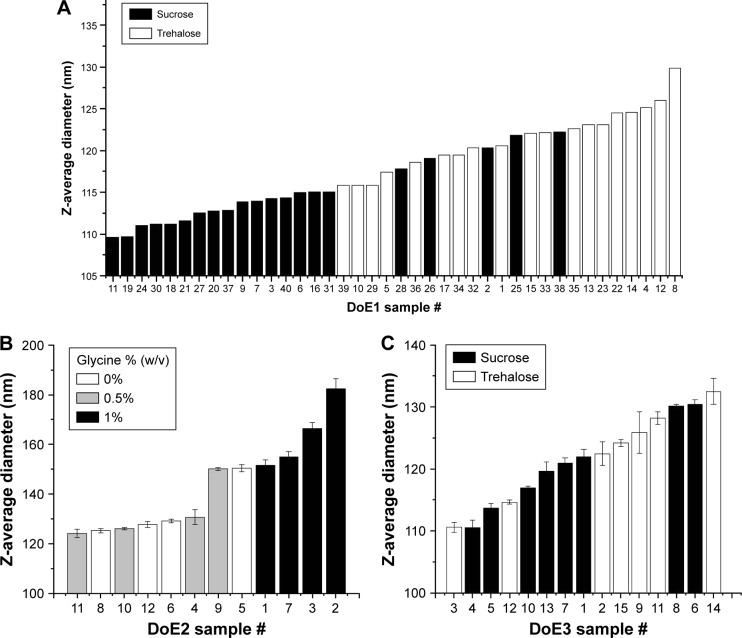
Z-average diameters measured by DLS for lyophilized and reconstituted samples from DoE1 (**A**), DoE2 (**B**), and DoE3 (**C**). Particle size measured after lyophilization and reconstitution. Trends between disaccharide type for DoE1 and glycine concentration for DoE2 are apparent. See Tables S2–S4 for sample compositions. **Abbreviations:** DLS, dynamic light scattering; DoE, design of experiments.

**Figure 8:**
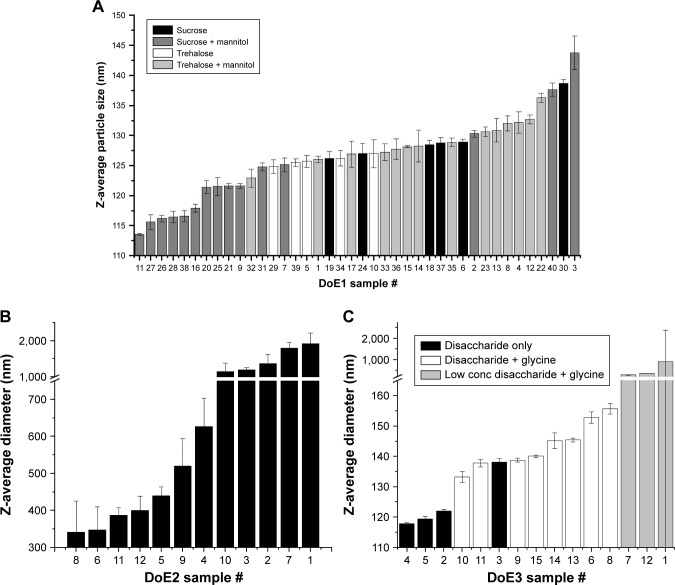
Z-average diameters measured by DLS for heat-stressed samples from DoE1 (**A**), DoE2 (**B**), and DoE3 (**C**). Sucrose–mannitol samples displayed the smallest particle size for DoE1. Particle size for DOE2 increased dramatically for all samples. Particle size increased for glycine-containing samples in DoE3. See Tables S2–S4 for sample compositions. **Abbreviations:** DLS, dynamic light scattering; DoE, design of experiments.

**Figure 9:**
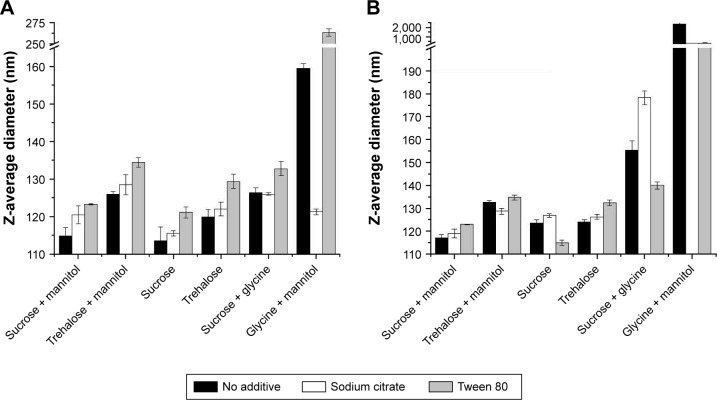
Z-average diameters measured by DLS for samples from the additive study after lyophilization (**A**) and heat stress (**B**). Sodium citrate decreased particle size for the glycine–mannitol formulation after lyophilization, but no benefit was seen for either sodium citrate or Tween 80 in other samples. See Table S5 for sample compositions. **Abbreviation:** DLS, dynamic light scattering.

**Figure 10:**
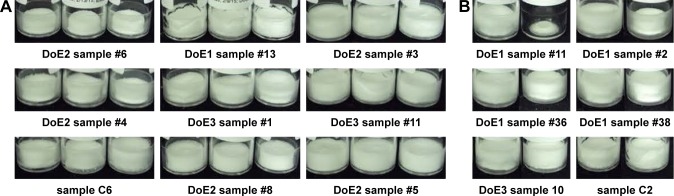
Examples of lyophilized cakes after lyophilization (**A**) characterized as poor (first column), acceptable (second column), or excellent (third column). Specific classification criteria are listed in the “Materials and methods” section. Examples of changes observed in lyophilized cakes after heat stress (**B**). The paired vial images indicate sample appearance before (left vial) or after (right vial) heat stress. Examples of major (top row), minor (middle row), and no change (bottom row) are shown. See Tables S2–S5 for sample compositions. **Abbreviation:** DoE, design of experiments.

Samples from the three DoE and the additive studies were analyzed after formulation, lyophilization, and thermal stress at 50°C for 30 days. Sample analysis included reducing SDS-PAGE,^29^ pH measurements, DLS, assessment of lyophilized cake quality, and HPLC-CAD to determine GLA concentration. No loss or apparent decrease in ID93 band intensity was observed on SDS-PAGE after formulation, lyophilization, or thermal stress, indicating that ID93 remains intact for all samples (data not shown). pH measurements, and Z-average diameter, GLA concentration, and cake quality assessments are summarized in Figures 6–10.

Figure 6 shows the change in pH for DoE1 samples after formulation, lyophilization, and thermal stress. General trends were apparent when pH changes were examined by buffer type and pH. All samples which contained Tris buffer decreased in pH after lyophilization; however, no consistent decrease was observed for phosphate-buffered samples. Samples from DoE2, DoE3, and the additive study all included Tris pH 8.0 buffer and exhibited a similar decrease in pH after lyophilization as DoE1.

For all formulations tested, the Z-average diameter after formulation was between 91.7 and 96.8 nm (data not shown), indicating minimal particle size increase over GLA-SE alone (91.6 ± 0.9 nm). After lyophilization and reconstitution, the particle size increased in all formulations. In DoE1, the Z-average diameter range was 109.6–129.8 nm and varied continuously across this range for this set of samples (Figure 7A). The 16 formulations with the smallest particle size contained sucrose, indicating that sucrose inhibited particle size increase resultant from lyophilization. In DoE2, larger Z-average diameters (124.1–182.3 nm) were observed than in DoE1 or DoE3, the distribution of sizes was discontinuous, and the largest particle size was observed for samples containing 1% (w/v) glycine (Figure 7B). Z-average diameters after lyophilization and reconstitution in DoE3 were similar to DoE1, though the influence of disaccharide type on particle size trends was less clear (Figure 7C). After thermal stress at 50°C for 30 days, the average diameter increased for DoE1 samples by ~9 nm, and the 10 samples with the lowest particle sizes all contained sucrose and at least 0.5% (w/v) mannitol (Figure 8A). A dramatic increase in particle size to at least 300 nm for all samples in DoE2 was seen, indicating that the glycine–mannitol formulations included in this study are not stable to thermal stress at 50°C (Figure 8B); however, the stability of these formulations at lower temperatures was not assessed in this study. Glycine similarly had a negative effect on particle size after thermal stress in disaccharide–glycine formulations: samples which contained glycine in DoE3 had a larger particle size than samples which contained disaccharide only, and samples which contained glycine and the lowest percentage of disaccharide (2.5%, w/v) had a particle size that increased to at least 275 nm (Figure 8C). For the additive study (Figure 9), the addition of sodium citrate resulted in smaller particle size for the glycine–mannitol formulation after lyophilization (120.5 nm, Figure 9A); however, no other benefits were observed by the inclusion of sodium citrate or Tween 80. Similarly, no substantial decrease in particle size after heat stress (Figure 9B) relative to samples without additives was apparent in any of the formulations tested.

GLA concentration did not significantly decrease below the expected 50 µg/mL after lyophilization for any of the samples studied; however, varied concentrations were obtained after heat stress. GLA was not detectable in any of the samples from DoE2 after heat stress (data not shown). In DoE1 and DoE3, a dependence on disaccharide type was observed (Figure S1), and sucrose was generally poorer at protecting against GLA degradation. In DoE1, the sucrose–Tris formulations had the lowest GLA content after heat stress compared to the other disaccharide–buffer combinations.

Cake quality immediately after lyophilization was assessed as described in the “Materials and methods” section and ranked as “excellent”, “acceptable”, or “poor”. After stress, cakes were then assigned a classification of “no change” indicating no change from post-lyophilization appearance, or “change” indicating noticeable minor-to-major changes. Figure 10 shows the representative images post-lyophilization. In general, the cakes can be described as pharmaceutically acceptable in appearance post-lyophilization (inclusive of “acceptable” and “excellent” rankings). Only a total of six cakes were ranked as “poor”, and these were observed in DoE2 and the additive study. After stress, many samples exhibited change originally categorized as pharmaceutically acceptable or poor post-lyophilization (Figure 10, Panel B). In DoE1, 10 out of the 40 samples exhibited change after heat stress, and the samples that changed were predominantly buffered by Tris pH 7.5 and contained sucrose with higher mannitol concentration (0.5%–1%, w/v). In DoE2, all samples underwent changes, most of them major changes, resulting in severely collapsed cakes that required up to 2 minutes for full reconstitution. This suggests that mannitol and glycine formulations, alone or in combination at the concentrations tested, are not thermostable at 50°C. In DoE3, two out of 15 samples experienced cake quality changes after heat stress. These samples were replicates containing 2.5% sucrose and 0.45% glycine in Tris pH 8. All other samples did not change, suggesting that sucrose can be formulated with glycine for pharmaceutically acceptable and thermostable cakes as long as sucrose content is sufficiently high. The additive samples, when excluding poor post-lyophilization cakes, included three samples exhibiting minor changes. The presence of Tween 80 or sodium citrate did not change the physical appearance or reconstitution times of samples before or after stress, with the exception of one sample: 1% glycine, 1% mannitol. For this formulation, which formed an “excellent” cake without additive, inclusion of either sodium citrate or Tween 80 resulted in cakes that were ranked “poor”; additionally, none of the 1% glycine, 1% mannitol formulations was stable after thermal stress.

In general, cakes that contained trehalose were superior to sucrose, and cakes buffered with sodium phosphate tended to be more thermostable than Tris. Glycine and mannitol can be used to form pharmaceutically acceptable cakes when in appropriate combination with a disaccharide, but should not be the sole excipient in a formulation if thermostability is desired. Lastly, the additives, Tween 80 and sodium citrate, did not improve the thermostability of the tested formulations.

Based on evaluation of the physicochemical data, further statistical evaluations of DoE1 and DoE3 were performed, and models were derived from fits of the experimental data that allowed prediction of responses across the range of factors evaluated in these studies. DoE2 yielded “excellent” cakes after lyophilization; however, they were not able to maintain particle size or GLA concentration after heat stress, so glycine–mannitol formulations were excluded from further analysis. Though glycine–mannitol formulations did not meet the thermostability criteria set for this study, these formulations may meet less stringent stability criteria. DoE response data were fit using a least squares model. Parameters for main effects, second- and third-order interactions, and second powers for continuous factors were included. Responses evaluated in the model were particle size after lyophilization, particle size after stress, GLA concentration after stress, the change in pH after lyophilization, the change in pH after stress, and cake quality.

The fit of the least squares model could be evaluated by comparing the measured (actual) response to the response predicted by the model for each sample. For DoE1 (Table S6), good linear fits were obtained for each response with *R*^2^ values for the actual-by-predicted plots between 0.87 and 0.97. Significant parameters for the fit to each response were obtained. Disaccharide type and buffer type were significant parameters for most responses. Overall, utilization of sucrose as an excipient resulted in a lower particle size after lyophilization and to a lesser extent after stress, but less resilience to GLA degradation after stress was observed. Sucrose samples had a slightly smaller pH change after stress. Buffer type was a significant parameter for cake quality, GLA concentration, and pH change upon lyophilization. Tris buffer reduced cake quality, was less resilient to pH change upon lyophilization, and did not protect against GLA degradation as well as phosphate buffer. Other significant parameters were pH, mannitol concentration, disaccharide concentration, and second-order interactions between factors. In detail, pH 7.5 protected against GLA degradation and pH change upon lyophilization better than pH 8.0. Inclusion of mannitol generally increased particle size upon lyophilization but had little independent impact on particle size after stress. However, the interaction between mannitol concentration and disaccharide type did impact particle size after stress. Increasing mannitol concentration reduced post-stress particle size with sucrose, but increased post-stress particle size with trehalose. The second-power disaccharide concentration parameter had a significant and negative effect on change in pH after stress, with an optimum concentration at 7% (w/v). Lastly, the buffer type × disaccharide concentration parameter was a significant parameter for GLA degradation. With Tris buffer, increasing disaccharide concentration protected against GLA degradation, but with sodium phosphate, disaccharide concentration did not affect GLA retention.

Optimized formulations were selected from DoE1 by evaluating the model using a set of desirability functions as described in the “DoE and analysis of results using JMP software” section. A desirability value of 1.0 indicates that all predicted responses match target desirability values, and a desirability value of 0 indicates that all responses are outside of the desirable range. Desirability values can be used comparatively to assess conditions predicted by the model. Table 3 outlines the optimized formulations for the eight possible combinations of disaccharide, mannitol, and buffer type combinations and their predicted responses. The overall optimum formulation is 6.8% (w/v) trehalose, 0.0% (w/v) mannitol, sodium phosphate pH 7.5, with a desirability score of 0.68, and the model predicts that higher trehalose concentrations up to 10% (w/v) do not adversely impact desirability (data not shown). The optimized trehalose–Tris formulation has a lower desirability score (0.63) due primarily to a predicted decrease in pH after lyophilization; however, the other predicted responses were similar to the overall optimum formulation, and stability to GLA degradation was slightly improved. The trehalose–mannitol formulations in either Tris or phosphate buffer had a lower desirability than without mannitol, and the trehalose–mannitol–phosphate formulation had a higher desirability than the trehalose–mannitol–Tris formulation (0.57 vs 0.48, respectively).

An optimized sucrose formulation contains 7.5% (w/v) sucrose, 1% (w/v) mannitol, sodium phosphate pH 8.0, and has a desirability score of 0.66. Varying mannitol concentrations between 0.5% (w/v) and 1% (w/v) had very similar desirability as did sucrose concentrations between 3.5% (w/v) and 10% (w/v) (data not shown). An optimum sucrose formulation without mannitol contains 5.7% sucrose, 0.0% mannitol, phosphate buffer pH 7.5, and a desirability score of 0.65. The optimum sucrose–Tris formulation without mannitol contains 10% sucrose, 0.0% mannitol, Tris pH 7.5, and has a desirability score of 0.54. Lastly, the optimized sucrose–mannitol–Tris formulation yields the lowest desirability (0.16) and is predicted to have low cake quality after heat stress.

DoE3 (Table S7) responses were also fit with a least squares model. For particle size after lyophilization, GLA concentration, and cake quality after stress, *R*^2^ values for the actual-by-predicted plots were between 0.93 and 0.97, indicating that the model adequately describes the data; however, lower *R*^2^ values were obtained for particle size after heat stress and both ΔpH responses (*R*^2^ = 0.65–0.74), suggesting that the model may not adequately describe the data for these responses, potentially due to the smaller sample size. In DoE1, disaccharide concentration and disaccharide type both influenced pH change after stress; however, for DoE3, no significance was found. GLA concentration was well fit, and significant parameters were identified (disaccharide concentration, disaccharide type, and disaccharide concentration-squared). For cake quality, five significant parameters were identified, and the relationship between cake quality and the factors studied depended on multiple interactions. Glycine increased particle size both after lyophilization and after stress. Increasing disaccharide concentration increased GLA stability, and trehalose better protected against GLA thermal degradation. Disaccharide concentration and glycine concentration had a combined effect on heat-stressed particle size. At low glycine concentrations, increasing disaccharide concentration decreased particle size, while at high glycine concentrations, increasing disaccharide concentration increased particle size. In trehalose, low disaccharide concentration had a more deleterious effect on particle size after lyophilization than in sucrose. Finally, stressed particle size for trehalose-containing samples was less sensitive to glycine concentration than sucrose-containing samples. The optimum formulation, based on maximizing desirability functions for all responses, was very similar to the no-mannitol, sucrose-containing optimum from DoE1: 8.7% sucrose, 0.0% glycine, desirability score 0.51. No combination of glycine and either sucrose or trehalose was superior to the optimum formulations from DoE1, indicating that the addition of glycine is not advantageous in achieving a thermostable formulation at 50°C; however, the stability of glycine–disaccharide formulations may still be acceptable at lower temperatures.

The physicochemical data collected for the three DoE studies and the additive study provide useful insight into the favorable or non-favorable inclusion of the six excipients in lead formulations. In general, the inclusion of Tween 80 or sodium citrate was not advantageous in maintaining particle size or GLA concentration after thermal stress. Additionally, the inclusion of glycine, while increasing the cake quality after lyophilization in glycine–mannitol formulations, resulted in samples with increased particle size, and cakes that showed significant collapse, lower GLA concentration after thermal stress, and displayed no benefit over disaccharide-only formulations. This led to the identification of disaccharide and disaccharide–mannitol formulations as the most promising formulations for ID93 + GLA-SE which were compared by stability testing.

Both trehalose and sucrose demonstrated beneficial results; however, the inclusion of mannitol decreased desirability for all disaccharide–buffer combinations with the exception of sucrose–phosphate formulations. This led to the identification of six formulation classes of disaccharide–mannitol–buffer with higher stability including trehalose–phosphate (TP), trehalose–Tris (TT), trehalose–mannitol–phosphate (TMP), sucrose–mannitol–phosphate (SMP), sucrose–phosphate (SP), and sucrose–Tris (ST) with desirability scores of 0.54–0.68 (Table 3). Each of these six formulations were predicted to yield a particle size of 111–122 nm after lyophilization and 119–131 nm after thermal stress, experience a 5%–35% loss of GLA after thermal stress, and to maintain a pharmaceutically acceptable cake, even after 30 days at 50°C. These lead formulations, with slight modifications to achieve more isotonic solutions after reconstitution, were subsequently evaluated in a storage stability study.

### Stability of lead formulations

**Figure 11:**
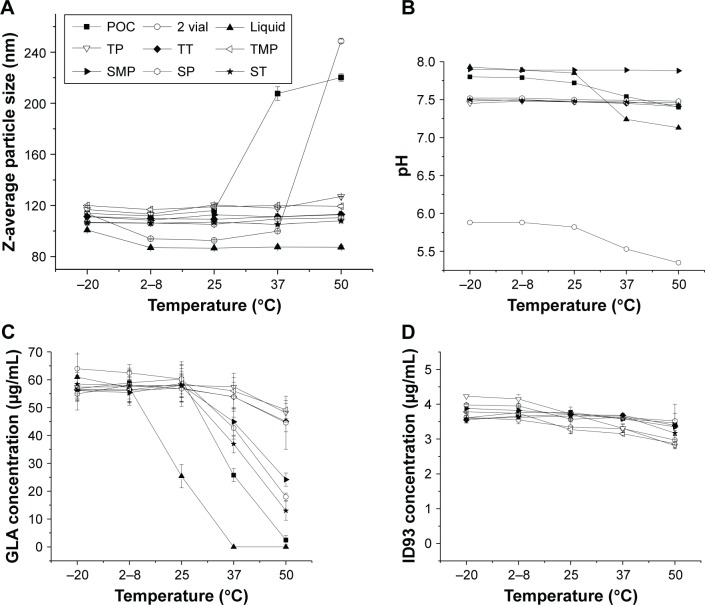
Z-average diameter (**A**), pH measurements (**B**), GLA content (**C**), and ID93 content (**D**) for samples stored for 3 months at the indicated temperatures. The six lead formulations (TP, TT, TMP, SMP, SP, and ST), the POC formulation, two-vial clinical presentation, and a liquid single-vial comparator are shown. Error bars indicate the standard deviation of two (**D**) or three (**A** and **C**) measurements. **Abbreviations:** GLA, glucopyranosyl lipid adjuvant; TP, trehalose–phosphate; TT, trehalose–Tris; TMP, trehalose–mannitol–phosphate; SMP, sucrose–mannitol–phosphate; SP, sucrose–phosphate; ST, sucrose–Tris; POC, proof of concept.

A study was designed to evaluate the stability of six lead lyophilized candidate formulations (TP, TT, TMP, SMP, SP, and ST). The six lead lyophilized formulations were compared to three other vaccine presentations, including a liquid single-vial presentation (liquid), the current two-vial clinical presentation consisting of a lyophilized protein vial and liquid GLA-SE, and the proof-of-concept (POC) single-vial lyophilized formulation. The POC formulation differed from the six lead formulations in both formulation components (20 mM Tris pH 8.0, 5% [w/v] trehalose) and utilized a less conservative lyophilization cycle. It has been previously shown to convey partial stability to ID93 + GLA-SE after storage at elevated temperatures.^24^ The goal of this study was to identify one or more formulations that convey stability after 3 months storage at 37°C as demonstrated by the following preset criteria: retention of greater than 80% of the chemical components (ID93 and GLA), and less than a 50% increase in particle size upon reconstitution. Data were collected during this study at five storage temperatures (−20, 2–8, 25, 37, and 50°C), and Figure 11 summarizes the data after 3 months storage.

At each temperature, samples were visually inspected, and vial images are shown in Figure S2. Lyophilized cakes appeared as expected based on the results of the DoE study and retained their appearance when stored at −20, 2–8, 25, and 37°C. Cakes stored at 37°C or lower for 3 months retained the appearances observed at t = 0 (data not shown for lower temperatures); however, changes were observed in the POC, SMP, and ST samples after 3 months at 50°C (Figure S2). At this temperature, the POC sample was significantly collapsed and appeared yellow, while the SMP and ST samples were less significantly collapsed and retained their white appearance. Reconstitution times of all samples were less than 1 minute and in general less than 30 seconds, with the exception of the POC after 3 months at 50°C, which required over 5 minutes for reconstitution.

Particle size was monitored by DLS to determine Z-average diameter and polydispersity index values. The Z-average diameter for GLA-SE prior to lyophilization was 89 ± 0.4 nm. After lyophilization, the trehalose (TP, TT, TMP) and sucrose (SMP, SP, ST) lead formulations increased to 117 ± 5 nm and 108 ± 2 nm, respectively. Z-average diameter after 3 months storage as a function of temperature is plotted in Figure 11A. For the POC sample, increased particle size was observed with storage at 37 and 50°C, and Z-average diameter increased to over 200 nm. In general, no clear increase in size was observed for the six lead samples, even after 3 months at 50°C. For storage at −20°C, only the liquid and two-vial samples demonstrated a particle size increase, and particle size increased to a similar extent observed for samples after lyophilization. This suggests that particle size increases seen after lyophilization are primarily due to the freezing step and that further exposure to low temperatures after lyophilization does not result in additional size increases. This suggests that lyophilized ID93 + GLA-SE is stabilized to both high- and low-temperature excursions.^30^ Changes in pH after storage for 3 months were measured (Figure 11B). For the six lead formulations, pH changes were less than 0.12 pH units; however, decreases of 0.2–0.8 pH unit were seen for the liquid, two-vial, and POC samples when stored at 37 or 50°C.

Component integrity was investigated for GLA, squalene, and ID93. High GLA retention was observed for POC, liquid, and lead formulations when stored at −20 or 2°C–8°C (Figure 11C). A decrease in GLA concentration of ~50% was seen for the liquid sample when stored at 25°C for 3 months. A similar 50% decrease was seen for the POC sample when stored at 37°C for 3 months. GLA retention after 3 months at 37°C was greater than 90% for four of the lead samples (TP, TT, TMP, SP), including all lead samples formulated with trehalose (Table 4). ID93 integrity was investigated by reversed-phase HPLC (Figure 11D). After 3 months at 37°C, similar ID93 retention was seen for the six lead samples and the POC with ID93 concentration ranging from 3.2–3.7 µg/mL. The greatest loss (21%) was observed for the TMP sample; however, all other lead samples lost between 8 and 17% at this temperature (Table 4).

Table 4 summarizes the results for the six lead formulations and POC sample relative to the preset stability criteria. For all six lead samples, the preset criteria were met, and superiority was demonstrated relative to the POC for maintenance of particle size. No clear increase in particle size was observed for the six lead formulations, whereas an 80% increase in Z-average diameter was observed for the POC sample. GLA retention of all six lead samples was superior to the POC: ~50% of the GLA was lost after 3 months for the POC. Four of the leads demonstrated a less than 10% loss in GLA content, and two lead formulations, SMP and ST, lost 20% and 36%, respectively. Interestingly, the leads that lost the most GLA and the POC all showed partial cake collapse after 3 months at 50°C, suggesting that there is a relationship between cake collapse and GLA retention. Additionally, these two formulations were predicted to be the most susceptible to GLA degradation based on the results of the model from the DoE study. ID93 loss was between 8 and 21% for the POC and lead formulations. Only one sample (TMP) was just outside of the preset criteria for ID93 retention. Based on these results, TT, TP, and SP have met the preset stability criteria.

### Immunogenicity of lead formulations

**Figure 12:**
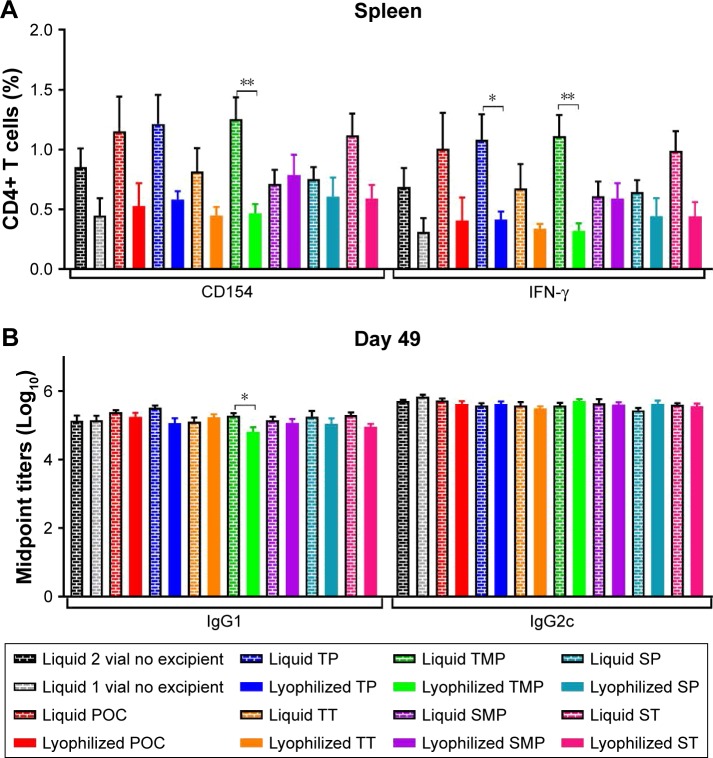
Cellular and antibody responses in mice immunized with control liquid formulations and the reconstituted lead lyophilized formulations. (**A**) CD4+ T cells collected from splenocytes 4 weeks after boost immunization were stimulated with ID93 and assessed for intracellular CD154 and IFN-γ production. (**B**) Serum antibody isotypes IgG1 and IgG2c were measured from sera collected 4 weeks following boost immunization. Data represent ten mice pooled from two independent experiments with five mice per experiment. Graphs show mean values ± SEM for each group. Statistics by two-way ANOVA with Dunnett’s correction for multiple comparison test relative to the control liquid two vial with no excipient and one-way ANOVA with Sidak’s correction for multiple comparison test within liquid and lyophilized pairs; *P < 0.05; **P < 0.01. **Abbreviations:** POC, proof of concept; TP, trehalose–phosphate; TT, trehalose–Tris; TMP, trehalose–mannitol–phosphate; SMP, sucrose–mannitol–phosphate; SP, sucrose–phosphate; ST, sucrose–Tris.

To investigate the effect of lyophilization of formulations on the immunogenicity of the vaccine, mice were immunized twice, 3 weeks apart, with ID93 + GLA-SE control liquid formulations as well as the reconstituted lead lyophilized formulations TT, TP, ST, SP, SMT, TMP. Four weeks after the second immunization, T cell and antibody responses were evaluated. We found a slight decrease in the antigen-specific CD4+ T cell TH1 response (ie, producing IFN-γ upon stimulation with ID93) toward most of the lyophilized formulations compared to their liquid form including the POC control (Figures 12 and S3). Nevertheless, no significant differences were found when compared to the control liquid two-vial formulation (consisting of the current clinical presentation of lyophilized ID93 that is reconstituted with water and mixed with liquid GLA-SE) indicating that neither the inclusion of the stabilizing excipients or the lyophilization and reconstitution process had a significant detrimental impact on the immunogenicity of the vaccines. Liquid ID93 + GLA-SE induces a TH1 response with little or no measureable TH17 or TH2 responses.^23^ In keeping with those previous findings, we did not detect IL-17A or IL-5 production by CD4+ T cells upon ID93 stimulation in any of the immunized cohorts (data not shown). In keeping with this TH1 bias, antibody responses were predominantly IgG2c and were comparable across all groups except for IgG1 production in the lyophilized TMP formulation group that was slightly decreased compared to the liquid formulation.

Next, in order to test resistance to thermal stress, the different lyophilized formulations and the liquid single-vial control (ie, liquid ID93 and liquid GLA-SE together in one vial) were stored for 3 months at −20, 4, 25, 37, or 50°C, and T cell and antibody responses were evaluated. When stored at 4 or −20°C during 3 months, all the lyophilized formulations performed similarly to the single-vial liquid control in terms of TH1 induction and skewing toward IgG2c with the lyophilized SMP formulation showing a significant increase in CD4+ TH1 responses at 4°C and the POC formulation at −20°C (Figure S4). After 3 months of storage at 25°C, the lyophilized samples elicited substantially greater frequencies of TH1 responses than the liquid single-vial control sample (Figure S4).

**Figure 13:**
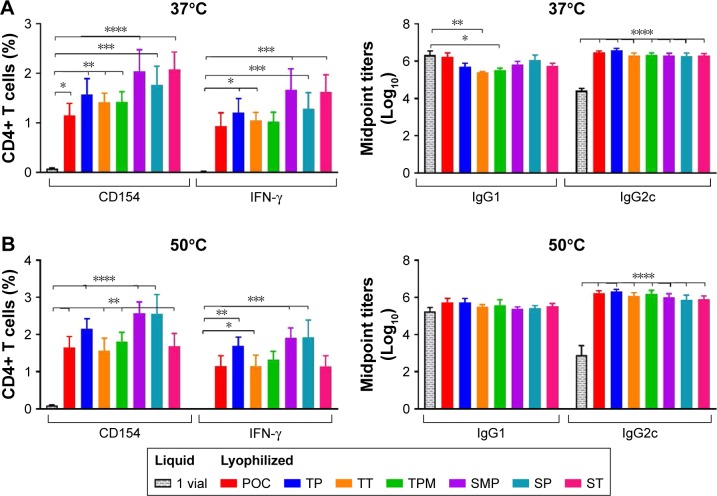
Cellular and antibody responses in mice immunized with formulations stored 3 months at 37°C (**A**) or 50°C (**B**). CD4+ T cells collected from splenocytes 4 weeks after boost immunization were stimulated with ID93 and assessed for CD154 and IFN-γ production (left panels). Serum antibody isotypes IgG1 and IgG2c were measured from sera collected 4 weeks following boost immunization (right panels). Data represent ten mice pooled from two independent experiments with five mice per experiment. Graphs show mean values ± SEM for each group. Statistics by two-way ANOVA with Dunnett’s correction for multiple comparison test relative to the control liquid single vial with no excipient; *P < 0.05; **P < 0.01; ***P < 0.001; ****P < 0.0001. **Abbreviations:** POC, proof of concept; TP, trehalose–phosphate; TT, trehalose–Tris; TMP, trehalose–mannitol–phosphate; SMP, sucrose–mannitol–phosphate; SP, sucrose–phosphate; ST, sucrose–Tris.

When exposed to elevated temperatures (37 or 50°C) for 3 months the liquid single-vial control formulation lost most of its immunogenicity as evidenced by the dramatic loss of CD4+ T cell and IgG2c antibody responses (Figure 13). In contrast, all the lyophilized formulation candidates were resistant to thermal stress as evidenced by the following: maintenance of frequency of ID93-specific CD4+ T cells (CD154); maintenance of IFN-γ-, TNF-, and IL-2-producing CD4+ T cells (Figures 13 and S5); no increase in IL-17A or IL-5 responses (data not shown); and maintenance of favorable IgG2c:IgG1 antibody titers in thermally stressed samples (Figure 13). These data indicate that lyophilization of a protein antigen with a TLR4 agonist and oil-in-water emulsion is beneficial to maintaining its biologic activity when exposed to prolonged elevated temperatures, which may have substantial benefits for the introduction and implementation of vaccines against pathogens such as *Mtb* that disproportionally affect the developing world where continuous cold-chain maintenance can be problematic.

## Conclusion

We utilized a DoE approach, informed by a preformulation study that selected stabilizing conditions and excipients, to identify six lead formulations of ID93 + GLA-SE that were predicted to be stable when stored at elevated temperatures. The six selected formulations were subsequently tested in an accelerated physicochemical stability study, and three formulations were identified that met our predefined stability criteria and demonstrated greater stability against GLA degradation and particle size increase than the previously described formulation.^24^ The identified formulations contained the following excipients: trehalose–Tris, trehalose–phosphate, and sucrose–phosphate. These formulations remained immunogenic (ie, inducing robust IgG2c antibodies and CD4+ TH1 cells) after lyophilization and heat stress for up to 3 months at temperatures up to 50°C. In summary, this work resulted in the development of three different formulations of ID93 + GLA-SE that demonstrated thermostability by maintaining physicochemical composition and immunogenic activity following extended thermal stress. Future studies will optimize the lyophilization process, identify a single formulation with optimal stability, and test the single-vial lyophilized product in clinical studies.

The broader implications of the results reported here include establishing the compatibility of the adjuvant, GLA-SE, with a wide range of pharmaceutical excipients. For instance, in many preclinical and clinical applications, it is typical for the adjuvant to be “bedside” mixed with the antigen proximate to immunization. Since GLA-SE demonstrated compatibility with most excipients tested when stored as a liquid for 24 hours, it is likely that GLA-SE would be compatible for bedside mixing with antigens that contain a variety of excipients. GLA-SE was also compatible with a range of excipients after lyophilization, including disaccharide, disaccharide–mannitol, disaccharide–glycine, and glycine–mannitol formulations. Thus, these compositions may be screened as platform formulations for other vaccines that contain GLA-SE or other similar nanoparticle adjuvants to more rapidly develop lyophilized presentations for adjuvanted vaccines. Thermal stability in the DoE study, however, was only achieved with select disaccharide or disaccharide–mannitol formulations. The heat stress condition utilized for the DoE study was storage for 30 days at 50°C, so it may be possible to identify stable glycine–mannitol or glycine–disaccharide formulations by studying less stringent conditions.

## Figures and Tables

**Table 1:** Maximum OD350 nm and Z-average particle size from the ID93 excipient screen and GLA-SE excipient compatibility studies

Excipient	Concentration	Max OD_350_^a^	Z-average_d_ t = 1 (nm)^b^	Z-average_d_ t = 24 (nm)^b^
No excipient	N/A	0.43 ± 0.02	91.5 ± 0.3	90.8 ± 0.4
Dextran sulfate	7.5 × 10^−6^ M	0.01 ± 0.02	125 ± 1	124 ± 1
Sodium citrate	0.01 M	0.04 ± 0.01	92.2 ± 0.4	91.9 ± 0.2
Brij 35	0.1% (w/v)	0.12 ± 0.00	92 ± 1	91.4 ± 0.8
Valine	0.20 M	0.12 ± 0.00	93.2 ± 0.9	92.9 ± 0.8
Threonine	0.30 M	0.15 ± 0.00	93.7 ± 0.2	92.1 ± 0.3
α-Cyclodextrin	2.5% (w/v)	0.18 ± 0.03	214 ± 2	241 ± 4
Histidine	0.15 M	0.20 ± 0.00	92.8 ± 0.7	93.0 ± 0.8
Glycerol	20% (w/v)	0.23 ± 0.03	95.0 ± 0.9	94.7 ± 0.7
Proline	0.3 M	0.23 ± 0.02	92.8 ± 0.5	92.4 ± 0.5
Mannitol	10% (w/v)	0.25 ± 0.01	94.2 ± 0.8	91.2 ± 0.5
Dextran T70	7.5 × 10^−6^ M	0.28 ± 0.02	91.7 ± 0.5	91.6 ± 0.3
Trehalose	20% (w/v)	0.28 ± 0.00	96 ± 1	95.5 ± 0.4
Tween 80	0.1% (w/v)	0.29 ± 0.01	92.2 ± 0.3	91.8 ± 0.8
Polyethylene glycol 6000	0.1% (w/v)	0.30 ± 0.00	91.4 ± 0.5	92.1 ± 0.9
Tween 20	0.1% (w/v)	0.31 ± 0.00	91.8 ± 0.4	91 ± 1
Sucrose	20% (w/v)	0.33 ± 0.01	95.6 ± 0.5	96.1 ± 0.9
Glycine	0.30 M	0.33 ± 0.01	93.1 ± 0.7	92 ± 1
Tween 20	0.001% (w/v)	0.35 ± 0.02	93.7 ± 0.7	94.3 ± 0.8
Lactose	7.5% (w/v)	0.36 ± 0.00	93.7 ± 0.5	93 ± 2
Asparagine	0.12 M	0.36 ± 0.02	92.6 ± 0.3	91.7 ± 0.2
Poloxamer 188	0.1% (w/v)	0.36 ± 0.01	92 ± 1	92.0 ± 0.7
Sorbitol	20% (w/v)	0.37 ± 0.00	96 ± 2	94.8 ± 0.8
Brij 35	0.001% (w/v)	0.42 ± 0.01	93.1 ± 0.2	93 ± 1
Tween 80	0.001% (w/v)	0.43 ± 0.00	93.9 ± 0.6	95.1 ± 0.6
Glutamic acid	0.05 M	0.83 ± 0.00	93.3 ± 0.7	90.3 ± 0.9
Aspartic acid	0.05 M	0.88 ± 0.01	92.1 ± 0.5	91.2 ± 0.8
Malic acid	0.15 M	0.96 ± 0.02	93.0 ± 0.6	91.7 ± 0.4
EDTA	0.20 M	1.00 ± 0.04	94 ± 2	93.8 ± 0.6
Sodium tartrate	0.15 M	1.02 ± 0.02	92.4 ± 0.2	93.2 ± 0.6
Glutathione	0.15 M	1.06 ± 0.00	93 ± 2	93.0 ± 0.4
Diethanolamine	0.30 M	1.10 ± 0.00	92.5 ± 0.4	92.4 ± 0.4
Guanidine	0.30 M	1.12 ± 0.00	94 ± 2	93.1 ± 0.7
Ascorbic acid	0.15 M	1.13 ± 0.01	93.7 ± 0.7	90.8 ± 0.5
Arginine hydrochloride	0.30 M	1.30 ± 0.03	94.4 ± 0.6	91.5 ± 0.6
Lactic acid	0.15 M	1.37 ± 0.02	93.0 ± 0.6	90.9 ± 0.8
Lysine	0.30 M	1.53 ± 0.02	94.2 ± 0.5	93.1 ± 0.4
Sodium chloride	0.9% (w/v)	1.61 ± 0.00	92.7 ± 0.7	91.2 ± 0.2

**Notes:**

a Maximum OD350 nm observed during kinetic turbidity-based excipient screening assay (Figure 5).

b GLA-SE Z-average diameter measured in the presence of the indicated excipient measured 1 or 24 hours after mixing.

**Table 2:** Factors used in DoE construction

Factor	Type	Range or factors
**DoE1, 40 samples**		
Mannitol concentration	Continuous	0–1% (w/v)
Disaccharide concentration	Continuous	3.5%–10% (w/v)
Disaccharide type	Categorical, 2 level	Sucrose, trehalose
Buffer type	Categorical, 2 level	Tris, sodium phosphate
Solution pH	Categorical, 2 level	7.5, 8.0
**DoE2, 12 samples**		
Mannitol concentration	Continuous	0–1% (w/v)
Glycine concentration	Continuous	0–1% (w/v)
**DoE3, 15 samples**		
Glycine concentration	Continuous	0–1% (w/v)
Disaccharide concentration	Continuous	2.5%–10% (w/v)
Disaccharide type	Categorical, 2 level	Sucrose, trehalose

**Abbreviation:** DoE, design of experiments.

**Table 3:** Optimized formulations and predicted responses, DoE1

Factor						Predicted response					
Desirability	Disaccharide concentration (%, w/v)	Disaccharide type	Mannitol (%, w/v)	Buffer	pH	Z-average_d_ (nm)^a^	Stressed Z-average_d_ (nm)^b^	GLA (μg/mL)^c^	ΔpH^d^	ΔpH stress^e^	Cake quality^f^
0.68	6.8%	Trehalose	0%	Phosphate	7.5	117	124	44	0	0	1
0.63	10%	Trehalose	0%	Tris	7.5	120	124	47	−0.2	0	1
0.57	8.4%	Trehalose	1%	Phosphate	7.5	122	131	43	0.1	0	1
0.48	8.2%	Trehalose	1%	Tris	7.5	124	132	40	−0.2	0	1
0.66	7.5%	Sucrose	1%	Phosphate	8	117	119	38	0	0	1
0.66	5.7%	Sucrose	0%	Phosphate	7.5	111	128	42	0	−0.1	1
0.54	10.0%	Sucrose	0%	Tris	7.5	112	127	33	−0.1	0	1
0.16	9.9%	Sucrose	1%	Tris	7.5	115	121	29	−0.2	0.1	2

**Notes:**

a Predicted Z-average diameter measured after lyophilization and reconstitution.

b Predicted Z-average diameter measured after heat stress for 30 days with storage at 50°C.

c Predicted GLA concentration after heat stress for 30 days with storage at 50°C.

d Predicted change in pH after lyophilization and reconstitution.

e Predicted change in pH after heat stress for 30 days with storage at 50°C.

f Predicted cake quality after heat stress for 30 days with storage at 50°C. All lyophilized cakes from DoE were considered acceptable after lyophilization and were designated 1 – no change or 2 – change based on visual observation after heat stress.

**Abbreviations:** GLA, glucopyranosyl lipid adjuvant; DoE, design of experiments.

**Table 4:** Stability comparison of lead lyophilized formulations vs POC samples after storage at 37°C for 3 months

Sample	Particle size change (<50%)^a^,^d^	GLA percent loss (<20%)^b^,^d^	ID93 percent loss (<20%)^c^,^d^
POC	84%	48%	10%
TP	0%	−2%	17%
TT	−1%	4%	8%
TMP	−1%	2%	21%
SMP	1%	20%	11%
SP	3%	7%	9%
ST	−2%	36%	9%

**Notes:**

a Z-average diameter change since lyophilization.

b GLA content change since t = 0.

c ID93 content change since t = 0.

d Preset stability criteria for storage at 37°C shown in parentheses.

**Abbreviations:** POC, proof of concept; GLA, glucopyranosyl lipid adjuvant; TP, trehalose–phosphate; TT, trehalose–Tris; TMP, trehalose–mannitol–phosphate; SMP, sucrose–mannitol–phosphate; SP, sucrose–phosphate; ST, sucrose–Tris.
